# Forecasting Multitrait Resistance Evolution under Antibiotic Stress

**DOI:** 10.1093/molbev/msag065

**Published:** 2026-03-13

**Authors:** Suvam Roy, Eric Libby, Peter A Lind

**Affiliations:** Department of Molecular Biology, Umeå University, Umeå, Sweden; Integrated Science Lab, Umeå University, Umeå, Sweden; Umeå Centre for Microbial Research, Umeå University, Umeå, Sweden; Integrated Science Lab, Umeå University, Umeå, Sweden; Umeå Centre for Microbial Research, Umeå University, Umeå, Sweden; Department of Mathematics and Mathematical Statistics, Umeå University, Umeå, Sweden; Department of Molecular Biology, Umeå University, Umeå, Sweden; Umeå Centre for Microbial Research, Umeå University, Umeå, Sweden

**Keywords:** antibiotic resistance, *Pseudomonas aeruginosa*, efflux pump, gene regulatory network, evolution, mathematical model

## Abstract

Many bacteria rely on efflux pumps to survive antibiotic stress and exposure to antibiotics often leads to mutations in pump genes or their regulators that increase pump expression. Predicting the spectrum of these mutations is important for designing effective antibiotic treatments, but the underlying regulatory networks are large and complex, making them difficult to map experimentally. To address this challenge, we developed a mathematical framework that integrates dynamical equations for efflux pump regulation with a genetic algorithm for parameter estimation and evolutionary simulations. Using this framework, we simulated in silico evolution of *Pseudomonas aeruginosa* under exposure to the antibiotics meropenem, tobramycin, and ciprofloxacin. The simulations revealed mutational spectra affecting the expression of four Resistance-Nodulation-Division efflux pumps and their shared regulatory network. The most frequently mutated genes were single-target regulators that matched well with previous observations in clinical and in vitro studies. The model also showed that the shared use of the OprM protein by two pumps is a key factor shaping their distinct mutational patterns. Mutations often produced multitrait phenotypes, manifesting as collateral sensitivity or cross-resistance to antibiotics not used for selection. While cross-resistance evolved readily, its extent depended on initial pump expression levels and thus may vary between strains. Finally, simulations of changing environments showed that efflux pump genes tend to be lost in the absence of antibiotics, suggesting a potential strategy to steer bacterial evolution toward reduced capacity to re-evolve resistance.

## Introduction

Bacteria regularly encounter toxic compounds, such as antibiotics, that threaten their survival. A common defense mechanism involves the use of efflux pumps that expel diverse toxic compounds from inside the cell (Piddock 2006). In addition to their roles in extruding toxic compounds, efflux pumps have major roles in bacterial physiology and metabolism and also impact biofilm formation and virulence (Whittle et al. 2024; Henderson et al. 2021). Accordingly, their production is normally tightly regulated and the regulators controlling these systems typically do not sense antibiotics directly, with the exceptions of dedicated systems such as the well-characterized TetR–TetA conferring inducible tetracycline resistance (Hillen and Berens 1994). In environments with antibiotics, bacteria often evolve multidrug resistance by acquiring mutations that cause overexpression of efflux pumps (Poole 2011; Costa et al. 2013). While predicting these mutations would be clinically useful, it is made difficult by the remarkably complex regulation of efflux pump systems (Weston et al. 2018; Wu et al. 2024).

Efflux pump expression is controlled by intricate hierarchical networks of both local and global transcription factors as well as post-translational and post-transcriptional regulation (Grkovic et al. 2002; Wu et al. 2024). A consequence of this complex regulation and the lack of direct sensing of antibiotics by regulators is that resistance mutations typically do not occur in the efflux pump genes themselves but rather in regulatory genes, which lead to constitutive overexpression and dysregulation of efflux pump production. Such dysregulation is expected to be costly in the absence of antibiotics, both because efflux pumps participate in vital cellular functions and because their overproduction imposes significant energetic and protein synthesis costs. These costs can drive secondary mutations in regulatory elements or even in the pump genes themselves (Marvig et al. 2014; Hernando-Amado et al. 2025), further degrading regulatory networks or resulting in partial or complete loss of efflux systems as a means of reducing fitness costs. Over short timescales, such as those encountered in laboratory evolution experiments or within-patient adaptation, such mutational trajectories might be used to steer populations toward low-fitness genotypes or toward loss of key resistance determinants. However, over longer evolutionary periods, such mutants are expected to be outcompeted or restored through recombination, given the fundamental importance of efflux pumps to bacterial physiology. Consequently, stable loss of efflux pump systems is not commonly observed in natural populations.

Predicting these mutational targets a priori and their functional consequences, eg fitness (Andersson 2006) or epistatic effects (Weinreich et al. 2006), is complicated by the complexity of interacting components. Moreover, once a mutation has fixed it can alter future mutational targets, having downstream effects on evolutionary trajectories. These effects can lead to medically relevant phenomena such as cross-resistance and collateral sensitivity (Lázár et al. 2013). Thus, predicting the effects of mutations on efflux pump regulation can have important implications for designing antibiotic treatments to pathogenic bacteria.

One way to develop and test such predictions is to carry out in vitro evolution experiments. The problem is that experimentally mapping this mutational landscape across entire regulatory networks is resource intensive (Toprak et al. 2011) due to the vast combinatorial space of possible mutations (Palmer and Kishony 2013), the need for precise genetic manipulation (Cui and Bikard 2016), and the requirement for high-throughput phenotypic screening under dynamic conditions. Consequently, computational and theoretical approaches coupled with evolutionary simulations (Lässig et al. 2017; Łuksza and Lässig 2014; Hermsen et al. 2012; Barton et al. 2016; Wang et al. 2015; Nourmohammad et al. 2016; Greulich et al. 2012; Siedentop et al. 2024; Pinheiro et al. 2021; Noble et al. 2017), can make important contributions. These theoretical frameworks have been shown to be useful in predicting mutational targets (Lind et al. 2019) and accounting for how the evolutionary history of a strain, ie previous mutations in regulators, can alter future resistance evolution. They are also highly adaptable to changes in gene content in different strains or changes in gene expression in different environments. Importantly, they can be used to search a large combinatorial space of mutations and simulate resistance evolution during sequential (Nichol et al. 2019) or combination (Gjini and Wood 2021) exposure to antibiotics, and thereby provide experimentally testable null models and possible targets for novel therapeutic strategies.

Developing a predictive theoretical approach requires a significant amount of empirical data. A single bacterial species can contain multiple efflux pump systems, each with their own regulators and specificity concerning which antibiotics they export. Since, the different efflux pumps also have cross-regulation, changes in the regulation of one pump may affect another. The requirement for empirical data to effectively model gene regulatory networks highlights the need for a well-characterized model system. To this end, *P. aeruginosa* is a good candidate because it is a clinically significant opportunistic pathogen that has been studied extensively. *Pseudomonas aeruginosa* possesses four major Resistance-Nodulation-Division (RND)-type efflux pumps linked to antibiotic resistance in the clinic: MexAB-OprM, MexXY-OprM, MexCD-OprJ, and MexEF-OprN (Li et al. 2016; Wu et al. 2024). Each pump comprises three components: two Mex proteins (localized to the inner membrane and periplasm) and one Opr protein (embedded in the outer membrane). An overview of the regulators of each pump and the antibiotics extruded by each pump is provided in Table 1. During antibiotic treatment, *P. aeruginosa* commonly evolves multidrug resistance through mutations in regulators, eg *nfxB* and *mexZ*, that lead to increased production of efflux pumps. Because of their role in resistance, many of the regulators have been subject to decades of experimental studies, providing a detailed mechanistic understanding of their functions. *Pseudomonas aeruginosa* therefore is an ideal model system to provide fundamental insights into the evolution of gene regulatory networks and phenomena such as cross-resistance and collateral sensitivity (Poole 2011).

**Table 1:** The genes related to the four RND efflux pumps in *P. aeruginosa* along with their targets (antibiotics or other genes).

Protein type	Gene(s)	Substrates	Reference
Efflux pump	*mexAB-oprM*	*β*-Lactams, fluoroquinolones,	Poole et al. (1993)
		Macrolides, and chloramphenicol	
	*mexXY-oprM*	aminoglycoside, tetracycline,	Mine et al. (1999) and Morita et al. (2012)
		Erythromycin, and cefepime	
	*mexCD-oprJ*	Fluoroquinolones, tetracyclines,	Jamal et al. (2023)
		and chloramphenicol	
	*mexEF-oprN*	Chloramphenicol, fluoroquinolones,	Frèdi Langendonk et al. (2021)
		Tetracycline, trimethoprim, and imipenem	
Regulator	*mexR*		Poole et al. (1996)
	*nalD*		Morita et al. (2006)
	*rocS2-rocA2*	*mexAB-oprM*	Sivaneson et al. (2011)
		*mexR*	
	*ampR*	*mexEF-oprN*	Balasubramanian et al. (2012)
		*mexAB-oprM*	
	*brlR*	*mexEF-oprN*	Liao et al. (2013)
	*armR*	*mexR*	Wilke et al. (2008)
	*nalC*	*armR*	Jeong et al. (2023)
		*mexR*	Dong et al. (2005)
	*vqsM*	*nfxB*	Liang et al. (2014)
		*mexCD-oprJ*	Purssell and Poole (2013)
	*nfxB*	*esrC*	
	*esrC*	*mexCD-oprJ*	
	*algU*	*mexCD-oprJ*	
		*esrC*	Purssell et al. (2014)
	*mexZ*	*mexXY*	Matsuo et al. (2004)
	*armZ*	*mexZ*	Kawalek et al. (2019)
	*suhB*	*armZ*	Shi et al. (2015)
	*rplU*	*armZ*	Lau et al. (2012)
	*amgS-amgR*	*mexXY*	Krahn et al. (2012)
		*mexXY*	Muller et al. (2011)
	*parS-parR*	*mexEF-oprN*	Wang et al. (2013)
	*mexT*	*mexEF-oprN*	Köhler et al. (1999)
	*mexS*	*mexT*	Sobel et al. (2005)
	*mvaT*	*mexEF-oprN*	Westfall et al. (2006)

Here, we modeled gene expression levels of *P. aeruginosa* efflux systems and their regulators following established kinetic modeling techniques (Karlebach and Shamir 2008). We developed a genetic algorithm to estimate the strengths of regulation and simulated the evolutionary trajectories under different antibiotic treatments. From our simulations, we identified commonly mutated genes and characterized the diversity of mutational pathways for three clinically relevant antibiotics from different classes (ciprofloxacin [CIP], meropenem [MER], and tobramycin [TOB]). We found that the presence of shared regulators and efflux pump components result in diverse patterns of cross-resistance and collateral sensitivity. Upon removal of antibiotics, we observed that resistant mutants acquired deleterious mutations in efflux pump genes as they were costly to produce and no longer useful. Our results provide a detailed analysis of how the complex regulatory networks associated with efflux pumps such as the one of *P. aeruginosa* might evolve under antibiotic treatments and produce experimentally testable predictions that might ultimately be useful for guiding clinical treatment regimes.

## Results

### Model overview

**Figure 1:**
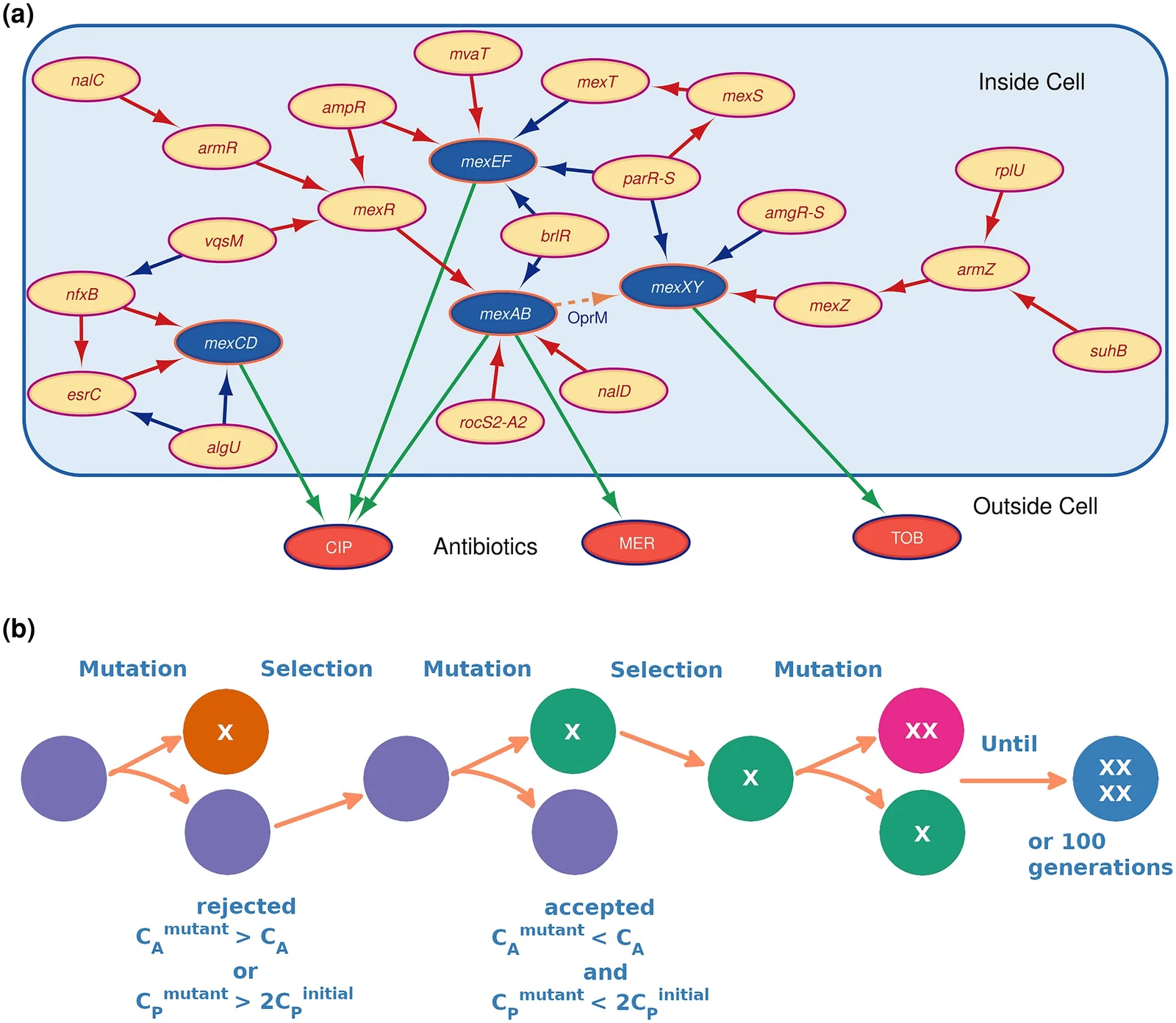
The regulation of efflux pumps and the evolutionary response to antibiotics. a) The diagram shows the gene regulatory network of four major RND efflux pumps in *Pseudomonas aeruginosa*. Blue nodes represent the efflux pump genes: MexAB (*mexA, mexB, oprM*), MexXY (*mexX, mexY, oprM*), MexCD (*mexC, mexD, oprJ*), and MexEF (*mexE, mexF, oprN*). Red nodes represent the three antibiotics we consider: CIP, MER, and TOB; and green arrows indicate which antibiotics each pump acts on. Yellow nodes represent regulatory genes with effects indicated by colored arrows: blue is up-regulation and red is down-regulation. The arrow labeled OprM is dashed to indicate that the MexXY pump requires OprM to be functional, and the *oprM* gene is transcribed together with *mexAB* in the *mexAB-oprM* operon. The regulatory proteins RocS2-A2 (RocS2-RocA2), AmgR-S (AmgR-AmS), and ParR-S (ParR-ParS) are two-component systems but in our model we consider them as single proteins. b) The schematic shows how evolution acts in our modeling framework in an environment with strongly inhibiting concentration of antibiotics. We start with a specific set of parameters describing the dynamics of the efflux regulatory network. Mutations occur randomly in regulatory genes and their benefit is assessed based on two criteria. First, they must increase resistance to antibiotics, represented in the model as lowering the cost of antibiotics ($C_{A}$). Second, they must not increase the total expression of protein (or cost of protein $C_{P}$) above some initial threshold, two times the initial $C_{P}$ in the model. This process is repeated until a total of four mutations are accepted or 100 mutations have been assessed.

The interactions in the regulatory networks for four efflux pumps in *P. aeruginosa* were defined based on experimental findings as described in Table 1 and shown in Fig. 1a. We described these regulatory interactions using a mathematical model composed of differential equations. In our model, the rates of transcription and translation were estimated from experimental data and the two processes were combined into a single-step (see “Protein concentrations inside cells” for details). The rates in our model pertaining to regulation however (strengths of regulation), are not known and difficult to measure experimentally. To find suitable strengths of regulation for our analyses, we instead fixed the steady state protein levels of the four efflux genes and used a genetic algorithm approach to identify regulation strengths that give rise to these steady states (see SI section “Genetic algorithm” for details). For the steady state protein levels of the efflux genes, we considered two different cases, A and B. In Case (A), we fixed the levels of all efflux pump proteins to the same value, 200, which is the average protein concentration per cell per gene in *P. aeruginosa*. In Case (B), we set the concentration of the MexAB pump to remain at average concentration (200) while the other pumps had lower levels (40) as expression levels are typically low for these pumps when not induced (Wurtzel et al. 2012).

Genetic algorithms have been used to find suitable parameters in other systems biology models (Moles et al. 2003; Fomekong-Nanfack et al. 2007; Fakhouri et al. 2010; Liberman et al. 2017; Parmar and Mendes 2019). Since they use a stochastic search process, genetic algorithms can potentially find different solutions to the same problem. For our application, this resulted in different strengths of regulation producing the same steady state concentrations of efflux pumps. We take advantage of this feature and run the genetic algorithm fitting process 25 times for each case, thereby producing a collection of possible regulatory models with the same phenotype (ie steady state efflux pump concentrations). The diversity of models and the effects of constraining the efflux protein levels can be found in Figs. S1, S2 and Supplementary section: Robustness of strength estimates.

Once we have an initial set of models we then simulate their evolution in an environment where inhibition by antibiotics is the dominant selection pressure. In our framework, adaptation occurs via mutations that cause deletions or duplications, which can also be interpreted as loss-of-function and gain-of-function mutations, respectively. For simplicity, we assume that mutations that increase fitness will fix while those that are neutral or impose a fitness cost are lost. The primary determinant of fitness in our system, when there is a strongly inhibiting concentration of antibiotics, is the internal antibiotic concentration of cells, which means that mutations that allow cells to more effectively pump out antibiotics will improve fitness. In the absence of any other determinant of fitness, mutations leading to increased production of efflux pumps would fix, regardless of whether antibiotics are present. To avoid such biologically unrealistic results, we add a constraint that mutations must not lead to substantial increases in total protein production, thereby enacting a type of energy budget (see Fitness costs for details of how antibiotic and protein production cost is calculated). Thus, our simulations represent evolutionary trajectories where each mutation increases the removal of antibiotics while not incurring large increases in protein production.

In our model, mutations occur randomly with an equal probability for deletion and duplication events (see Fig. 1b). Each evolutionary trajectory stops when either four mutations have fixed or 100 mutations have been assessed. This choice was inspired by typical treatment durations in the clinic and in vivo and in vitro experiments and thus represents short term evolution in the range of weeks to a few years. For each initial model (set of regulation strengths) we simulate 25 evolutionary trajectories.

### Mutational patterns and diversity

**Figure 2:**
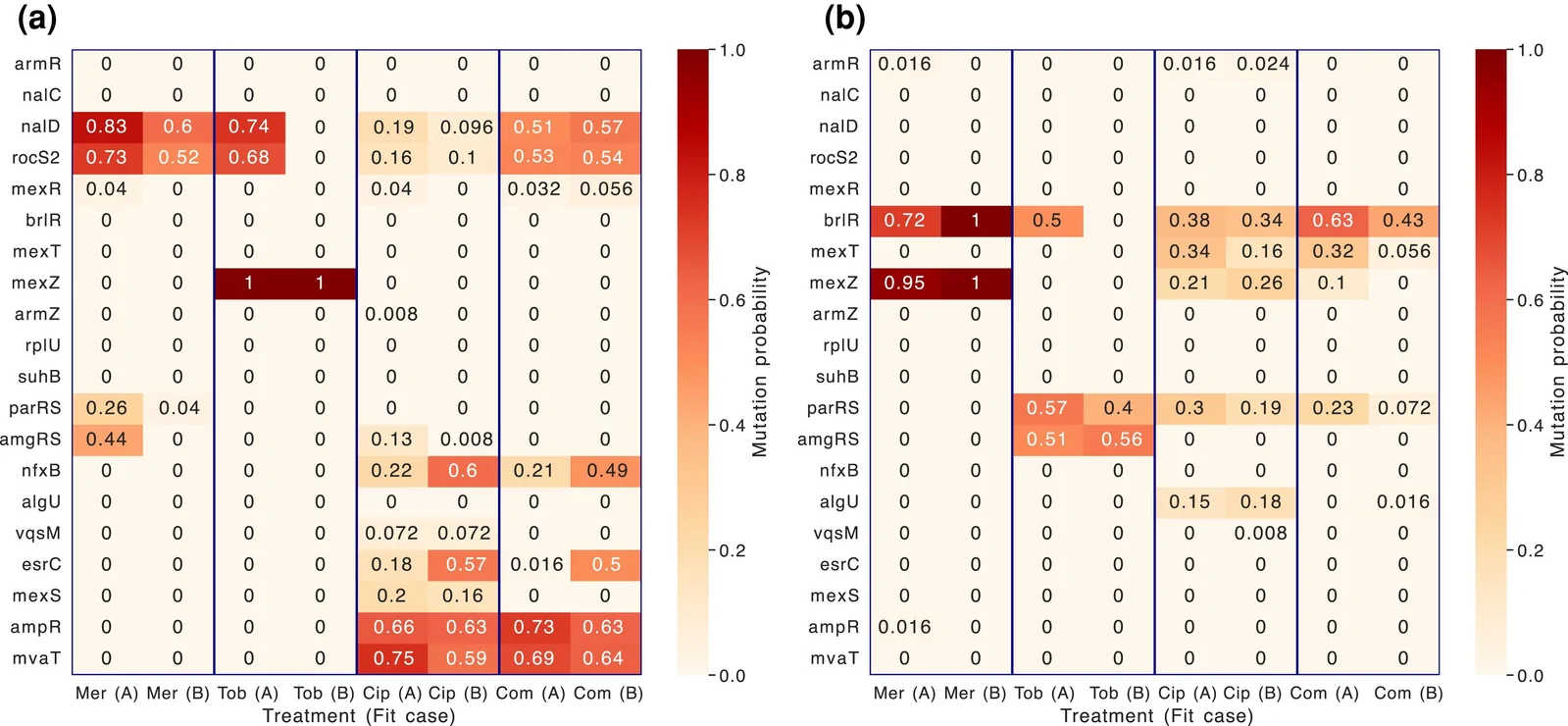
Mutation frequencies of regulatory genes, under different antibiotic treatments. a and b) Heatmaps show the frequencies of mutations of different regulatory genes, when evolved in different antibiotic treatments: meropenem (Mer), tobramycin (Tob), ciprofloxacin (Cip), or Com (a combination of all three). a) Heatmap shows deleterious or loss-of-function mutations while the a) heatmap shows gene duplication or gain-of-function mutations. The A and B labels after each antibiotic correspond to the two expression patters considered, ie Case A when all four pumps have same expression levels and Case B when the MexAB pump has a higher expression level. The mutational spectra for the two expression cases are largely similar. For both deletions and duplications, regulators with single targets tend to have higher mutation frequencies. The competition between MexAB and MexXY for OprM is an important determinant of the type of mutation. This competition results in deletion of *parRS* and *amgRS* and duplication of *mexZ* under MER treatment, while we observed opposite trend under TOB treatment.

As a result of our evolutionary simulations, we can determine the probabilities of deletion and duplication for all regulators in our model (see Fig. 2). We first describe mutations that confer resistance to MER. Since MER is only pumped out by MexAB, the most common mutations affect regulators of MexAB, ie deletions of its negative regulators *nalD* and *rocS2-A2* and duplications of its positive regulator *brlR*. Another frequent target is *mexZ* which occurs due to a competitive interaction between the two pumps MexAB and MexXY, the main pump for TOB: both pumps need OprM in order to be functional and OprM is expressed along with MexAB. Because MexXY competes with MexAB over OprM, increased MexAB functionality can be achieved by lowering the expression of MexXY, either by duplicating its negative regulator *mexZ* or deleting its positive regulators *parR-S* and *amgR-S*. The deletion of *parR-S* and *amgR-S* is observed only for the Case (A) when both pumps have the same initial expression level, because it lowers the expression of MexXY further.

Since TOB is pumped out by MexXY, selection under TOB treatment leads to the opposite mutations as in MER, ie deletions of *mexZ* or duplications of *parR-S* and *amgR-S*. Deletions of *mexZ* occurred in all simulations because it directly leads to increased expression of MexXY. Whether duplications of *parR-S* and *amgR-S* were found depended on the initial steady state concentration of MexXY, ie either Case A or Case B. Duplications of *parR-S* and *amgR-S* are more likely to occur in Case A than Case B, because the strength of these two regulators is low in Case B based on their kinetic parameters, thereby reducing their impact. Resistance to TOB also occurs via mutational targets *nalD* and *rocS2-A2*, but only after *mexZ* deletions. The reason is that MexXY needs OprM to form a functional efflux pump, and the *oprM* genes is transcribed from the *mexAB-*oprM** operon. So increasing the amount of MexAB leads to more OprM and thus more functional MexXY. For the same reason, we found duplications of *brlR* which upregulates MexAB, but only in Case A.

CIP is pumped out by MexAB-OprM, MexCD-OprJ, and MexEF-OprN. Therefore, we found deletions in the negative regulators of these pumps: *nalD* and *rocS2-A2* for MexAB-OprM; *nfxB* and *esrC* for MexCD-OprJ; and *ampR* and *mvaT* for MexEF-OprN. Of these negative regulators, the most common mutational targets were the *mvaT* and *ampR* genes. Both of these regulate MexEF-OprN directly and are relatively isolated in the regulatory network. Additionally, AmpR also regulates MexAB-OprM indirectly through MexR (see Fig. 1). Thus, mutating them can directly lead to increased MexEF-OprM production without any epistatic interactions. In terms of duplications, we found mutations in the positive regulators of pumps: *brlR* (for MexAB-OprM and MexEF-OprN), *mexT* and *parR-S* (for MexEF-OprN), and *algU* (for MexCD-OprJ). Since MexAB-OprM can pump out CIP we also found duplications in *mexZ*, so as to reduce the production of the competing pump MexXY.

In a combination treatment with all three antibiotics, we observe a similar mutational spectrum as with the CIP treatment, where multiple pumps are involved. We performed hierarchical clustering (Fig. S3) of these mutated genomes to determine the mutational diversity following each treatment. This figure indicates that the cells have only a few ways to become resistant under MER and TOB treatment, whereas, they can become resistant to CIP through a variety of different mutations. Also, the mutational diversity is lower in Case B versus Case A, when the MexAB pump has a higher expression level compared to the other pumps initially.

To check whether our sample size of 25 initial sets is sufficient to estimate mutational targets, we generated 100 sets for Case (B). We then randomly sampled 10, 25, 50, 100 sets from those 100 sets and calculated the mutation frequencies for one treatment. Figure S4 supports that 25 initial sets are a sufficient sample size, as the differences in mutation frequencies become negligible for even larger sample sizes. We also constructed two alternate versions of Case (B), A1: MexAB=333,MexXY=MexCD=MexEF=67 and A2: MexAB=300,MexXY=MexCD=MexEF=200. Figure S5 compares these two alternate versions with the original choice for Case (B) and shows that the mutational frequencies largely remain consistent (with some deviations being caused by changing the initial levels).

We also wanted to explore the effect of initial parameter sets on the observed mutational frequencies. Figure S6 shows the mutational frequencies for the MER treatment case for 25 trials of each 25 different sets of parameters. We find that our mutational forecasts are generally robust with respect to the initial parameter sets, though there are some differences. For instance, in 12 of the 25 parameter sets, mexR was frequently deleted but in the other 13 parameter sets it was never deleted. We investigated what drives such differences by comparing boxplots of the regulatory strengths where mexR is mutated (blue) versus not mutated (red) (see Fig. S7). While most regulatory links have similar distributions of strengths, the four links involving MexR have the most significant differences. Specifically, the strengths for regulators acting on MexR are higher for the mexR-nonmutated (red) sets. Conversely, the strength for MexR repressing MexAB, is lower in these sets. As all four interactions are negative regulations, this configuration of parameters makes MexR a weak repressor overall, and thus a less impactful mutational target. Therefore, while mutation probabilities are generally consistent, differences arise from plasticity in the regulatory network, highlighting the need for additional constraints to identify empirically relevant parameter sets.

### Collateral sensitivity and cross-resistance

**Figure 3:**
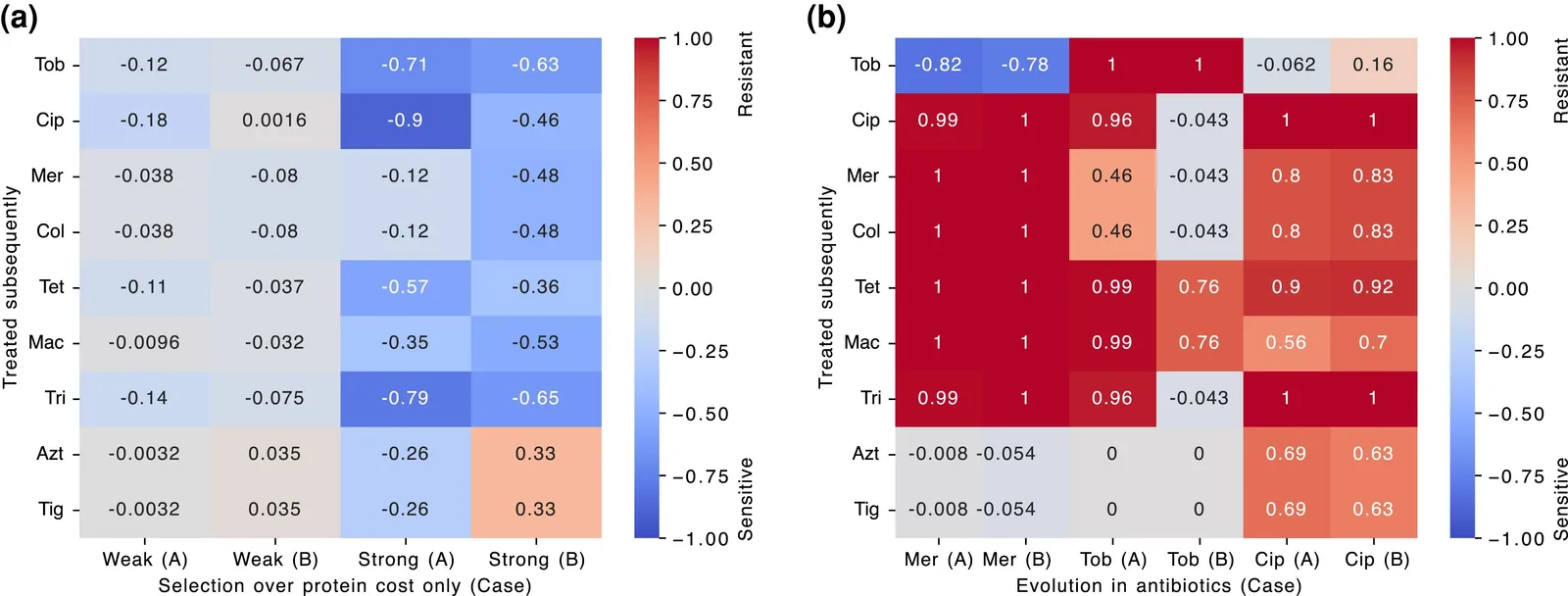
Evolution of cross-resistance and collateral sensitivity between different antibiotics. a) This heatmap shows the fraction of mutated genomes that become sensitive (blue) or resistant (red) to nine different antibiotics, when evolved without antibiotics. It also shows conditions of weak and strong selection over protein production costs. Evolution without antibiotics leads to bacteria becoming sensitive to most antibiotics, more so under strong selection over protein production cost. b) This heatmap shows the fraction of mutated genomes that become sensitive (blue) or resistant (red) to different antibiotics, when evolved in three different antibiotics. Adaptive evolution under antibiotic treatments (when protein cost becomes less important) leads to emergence of cross-resistance between different antibiotics for most cases. A notable case of collateral sensitivity is the enhanced susceptibility to TOB following MER treatment. In these heatmaps Case (A) and Case (B) denote the two cases of pump expression levels considered.

Using the mutated genomes from the three antibiotic treatments, we then assessed whether there were instances of multitrait phenotype evolution ie mutants evolved under one antibiotic having increased/decreased susceptibility to other antibiotics. This is commonly known as collateral sensitivity or cross-resistance respectively. For this analysis, we considered six more antibiotics: colistin, tetracycline, macrolide, trimethoprim, azithromycin, and tigecycline that can be extruded by one or several of the four efflux pumps. As a point of comparison, we also carried out mutation simulations in the absence of selection for antibiotic resistance, ie where selection only acts to constrain the cost of protein. We considered two constraints on protein costs for evolution in absence of antibiotics: (i) weak selection where the maximum amount of protein must not exceed twice the initial amount and (ii) strong selection where the amount of protein must always decrease after every mutation. Figure 3a shows that under weak selection for protein cost, the mutated genomes are about equally likely to become susceptible and resistant to the nine antibiotics. However, under strong selection, the mutated genomes become susceptible to a majority of the antibiotics. This is caused by the fact that each pump consists of multiple genes that contribute more to the protein production cost. Therefore, under strong selection, mutations that lower the expression of all pumps get preferentially selected, increasing the susceptibility of the cells to antibiotics. The notable exception here is the resistance to azithromycin and tigecycline, for the mutants generated in Case B. Due to the higher expression of the MexAB pump in Case B, a common mutation is deletion of *vqsM*, which causes upregulation of the MexCD pump that pumps out these antibiotics.

Next, we considered the possibility of cross-resistance and collateral sensitivity in genomes that evolved under selection for antibiotic resistance. We used the mutated genomes from the TOB, MER, and CIP treatments and checked their susceptibility against all nine antibiotics, ie the three used for selection plus six additional ones. Figure 3b shows that we find cross-resistance in all treatments and cases. For example, selection for resistance to MER resulted in cross-resistance to five other antibiotics. This increased propensity for cross-resistance is caused by a combination of factors: (i) regulators are shared between different pumps; (ii) antibiotics can be targeted by multiple pumps; and (iii) the same pump can act on multiple antibiotics.

We also found one case in which collateral sensitivity was prominent. The majority (around 80%) of genomes mutated under MER become susceptible to TOB. This collateral sensitivity is another consequence of the competition between the pumps MexAB and MexXY for OprM. Evolution of MER resistance often results in reduced expression of the MexXY pump, which makes the mutated genomes susceptible to TOB.

There is also a notable difference in sensitivity for the genomes evolved in TOB, between Case A (equal expression of all pumps) and Case B (higher expression of MexAB). For Case A, duplications of *parR-S* and *brlR* are common in TOB resistant mutants, which also upregulate the pumps MexAB-OprM and MexEF-OprN. These duplications causes cross-resistance to other antibiotics that are pumped out by those pumps. Since these duplications are not commonly found in Case B, there is less cross-resistance.

### Reversion of resistance

In our simulations cells evolve antibiotic resistance by increasing the expression levels of efflux pump genes. While this is beneficial in the presence of antibiotics, this imposes a cost once antibiotics are removed. Besides the cost of making pump proteins, it also costs energy to keep pumps running—though this running cost is not included in our model. Therefore, we were interested in whether mutants could evolve to the original level of efflux pump expression once antibiotics were removed from the system. We selected the mutants with the highest resistance to antibiotics—those paying the highest protein cost—from each initial set of estimated strengths of regulation. We then carried out evolution simulations in which mutations were kept provided that they reduced the amount of pump proteins. This selection criteria is similar to the strong selection from Fig. 3b except we disregarded the costs of regulatory proteins.

**Figure 4:**
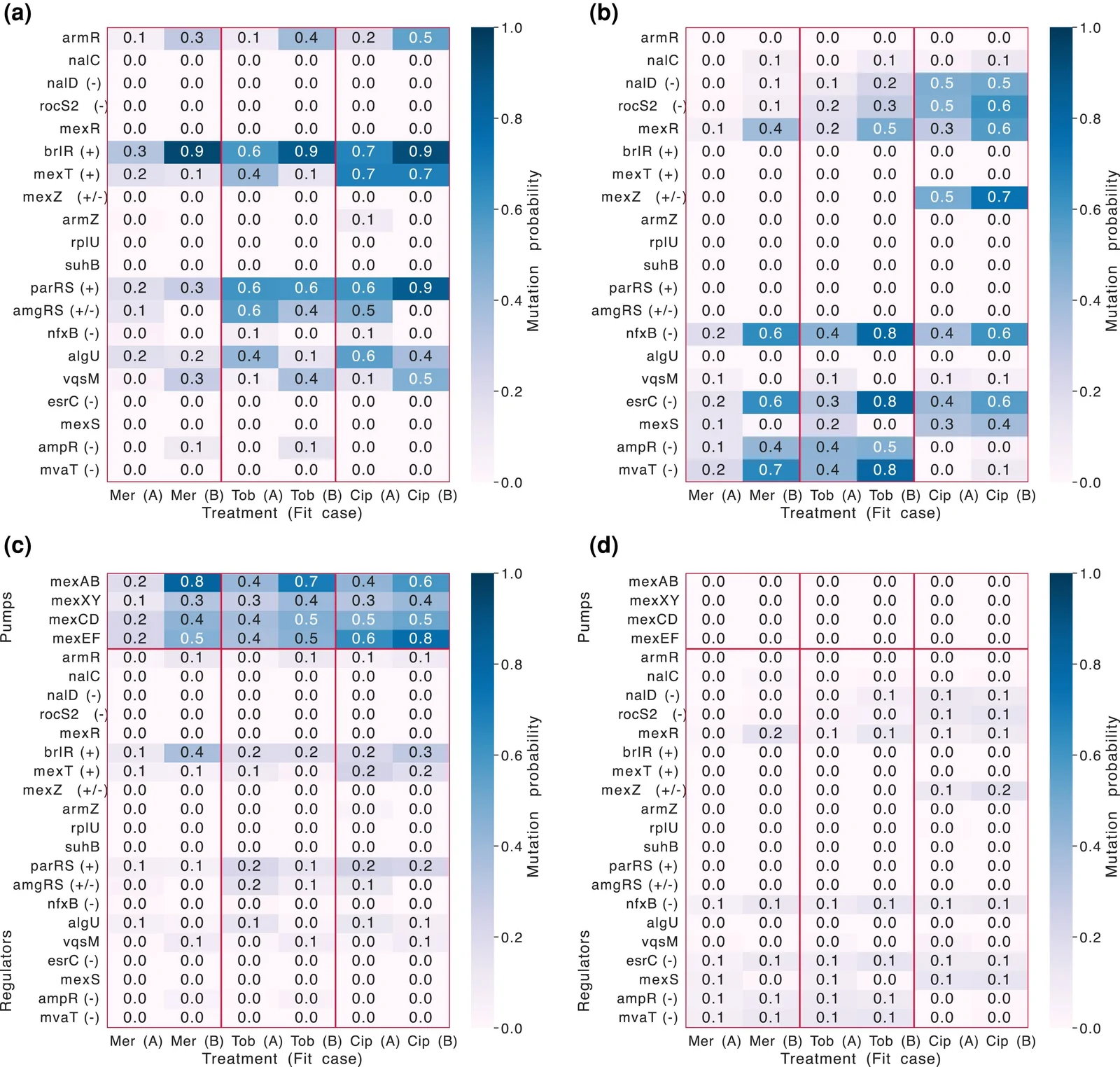
Mutation frequencies for resistant mutants when the antibiotics are removed. a and b) These heatmaps show the deletion and duplication frequencies respectively for the regulatory genes, when the fittest mutants are evolved without antibiotics. The (+), (−), and (+/−) denote genes that have been previously duplicated, deleted, or both duplicated and deleted depending on the antibiotic used. Reversion of resistance requires more duplications, compared to gaining resistance (see Fig. 2b). c and d) These heatmaps show the deletion and duplication frequencies respectively for both efflux pump and regulatory genes, when the mutants are evolved without antibiotics, while allowing for mutations in the efflux pumps genes as well. When the antibiotics are no longer present and the pump genes are also allowed to mutate, the most likely scenario is deletion of the pump genes. Case (A) and Case (B) denote two different cases of estimated strengths of regulation.

We observed that cells can evolve to lower efflux pump levels, but only in Case A could they return to the original levels prior to antibiotic exposure (see Fig. S8). Figure 4 shows the frequencies of different mutations that led to reduced pump expression. A comparison with Fig. 2 indicates that reducing the cost of pumps in the absence of antibiotics requires duplication of regulatory genes (negative regulators) at a higher frequency. But, as evident in Fig. S8, even these mutations are not enough to reach the original pump levels for Case B. This happens in Case B and not Case A because the negative regulators of MexXY, MexCD and MexEF are stronger. Once the cells lose those regulators via deletions, there is no way to get them back, and hence it is difficult to return to the original pump expression levels. These results demonstrate that in order to stop paying the extra costs of efflux pumps in the absence of antibiotics, it is easier to have deleterious mutations in the efflux pumps genes themselves rather than the regulators. We verified this when we allowed mutations in the efflux pump genes of the mutants (see Fig. 4).

### Adapting the model to new conditions

**Figure 5:**
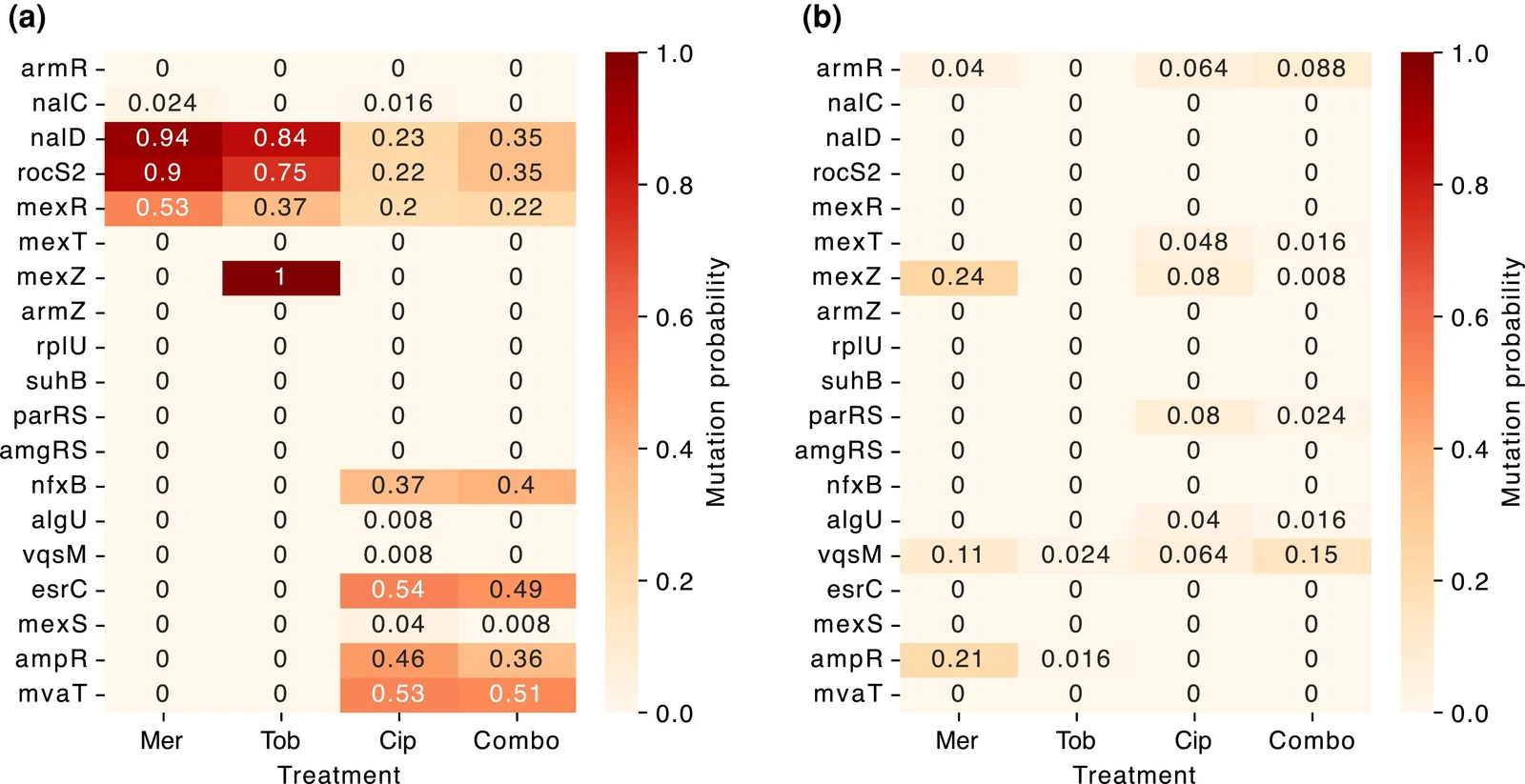
Mutation frequencies of different regulatory genes in the planktonic state. a) This heatmap shows the frequencies of deleterious mutations of the regulatory genes, under different antibiotic treatments. Making *mexR* a strong regulator in the planktonic state increases its deletion frequency significantly, compared to the biofilm state. b) This heatmap shows the frequencies of gene duplication mutations of the regulatory genes, when evolved in different antibiotics. The duplication frequency of *mexZ* is significantly reduced in the planktonic state. The planktonic state is only simulated for Case B, when the MexAB pump has higher expression compared to the other pumps.

*Pseudomonas aeruginosa* strains have differences in the genomic content of efflux components and regulators, as well as which genes are expressed—or proteins active—in particular environments. For example, the BrlR protein is a cyclic dimeric guanosine phosphate (c-di-GMP) responsive transcriptional regulator that is active only in cells with high levels of c-di-GMP, like those in biofilms, which is the context modeled in this article. To simulate evolution in the planktonic state, we removed the *brlR* gene from the network. Moreover, we can include experimental data from in vitro laboratory experiments to better fit parameter values. One example of this is *mexR* which is frequently mutated in planktonic experiments when adapting to CIP or MER (Barbosa et al. 2020; Ramsay et al. 2021) In our modeling of the biofilm context *mexR* rarely gets mutated, because the parameter fitting often leads to it being a weak repressor of the MexAB-OprM pump compared to other regulators such as *nalD* and *rocS2-A2*. We can take into account the experimental data and constrain the parameter fitting to cases in which MexR is a stronger regulator. After doing this, we simulated the planktonic state for Case B, where MexAB has a higher expression compared to other pumps. Figure 5 shows that these modifications led to an increased frequency of *mexR* mutations.

Another notable difference of evolution in the planktonic states compared to biofilms is the rarity of duplications. The gene *mexZ* was a frequent target of duplication in the biofilm state when exposed to MER, found in 95% to 97% of simulations. Its frequency is reduced to <25% in the planktonic state. As a result of this, there are fewer instances of collateral sensitivity arising from the MexXY pump (45% and 55% of cases are cross resistant and collaterally sensitive, respectively).

## Discussion

The ability to forecast and steer short-term evolution is of fundamental biological interest and has many potential applications especially in infectious diseases and cancer (Wortel et al. 2022). In addition to the inherent stochasticity of mutations, the complexity of gene regulatory networks often results in mutations having epistastic and pleiotropic effects that make predictions of evolutionary trajectories infeasible. Forecasting the evolution of antibiotic resistance through de novo mutations is a particularly suitable approach. Its advantages include the ability to fine-tune selection pressure via antibiotic concentration, well-studied resistance mechanisms, and partly characterized multitrait effects—where resistance to one antibiotic often increases or decreases resistance to others. Here, we modeled the gene regulatory networks of four efflux pumps and carried out simulations of mutational patterns in the presence and absence of antibiotics. This allowed us to explore the vast combinatorial space of mutations in a complex network and predict cross-resistance and collateral sensitivity after evolution in the presence of three clinically relevant antibiotics. We showed that under CIP treatment, the bacteria can become resistant in many different ways while for TOB and MER mutations were targeted to specific regulatory genes. In all treatments, we found repeated patterns: deletions of direct negative regulators and duplications of direct positive regulators. The mutants were multitrait in the sense that each mutant had reduced/enhanced susceptibility to multiple antibiotics and differing protein costs. So, based on the mutated genomes under these three different antibiotic treatments, we identified the emergence of cross-resistance and collateral sensitivity between these and six more antibiotics. We also showed that when antibiotics are removed, mutants acquire deletion mutations in efflux pump genes to reduce protein expression.

In our modeling framework, we represent mutational changes in regulatory genes and efflux pump components using two discrete categories: duplications, implemented as 2-fold increases in protein concentration, and deletions, corresponding to complete loss of the protein from the network. Gene duplications can also be interpreted as gain-of-function mutations that enhance regulatory strength but also incur an associated cost. In contrast, deletions represent general loss-of-function mutations that eliminate both regulatory activity and protein production costs, and, importantly, cannot be reversed by mutation within the model. Although additional mutational classes could in principle be incorporated, their effects are difficult to interpret and are insufficiently constrained by empirical data. For example, modeling a continuous distribution of mutational effects that produce subtler changes in protein concentration would generate many mutational trajectories that are functionally redundant, such as multiple partial loss-of-function mutations within the same gene. Similarly, allowing mutations to change binding affinities rather than protein abundance would introduce cost-free changes and possibilities for multiple gain-of-function mutations in the same gene. Another complication is that most deep mutational scanning studies do not distinguish between changes in protein abundance and alterations in molecular function. More complex evolutionary innovations, such as acquisition of new regulatory interactions or direct sensing of antibiotic concentrations by regulators, could also be modeled, but these are considered improbable and are not supported by empirical observations. For these reasons, we restrict the mutation landscape to duplications and deletions, which capture major functional transitions while remaining interpretable and grounded in available empirical evidence.

Our model can serve as a null framework for rapidly generating testable forecasts, even when experimental data on regulatory strengths or protein concentrations are lacking. One way of evaluating the accuracy of these forecasts is by comparing them with available mutational data from clinical settings or in vitro experiments. Since *P. aeruginosa* often evolves multidrug resistance during chronic lung infections in people with cystic fibrosis, genomic data have been collected on mutations arising under prolonged treatment with multiple antibiotics (Marvig et al. 2014). From these data, we see similar mutational targets as predicted by our model. For example, *mexZ* is one of the most commonly mutated genes in the clinic (Marvig et al. 2014), and it occurs in over 95% of our simulations in response to MER and TOB. Many additional genes in our model are also found to be mutated multiple times in the clinic, including *nfxB*, *mexT*, *mexS*, *parS*, *nalC*, *mexR*, *nalD*, and *rocS2*, and promoter mutations upstream of *esrC* and *rplU*. We can also make comparisons with a wealth of in vitro data, owing to *P. aeruginosa* being a model organism for antibiotic resistance. Similar to our simulations, adaptive in vitro evolution to CIP results in mutations of genes encoding negative regulators including *mexR*,*nalC*, *nalD*, *nfxB*, and *mexS* (Ahmed et al. 2020; Wardell et al. 2019). Resistance mutations in response to MER have been found in *nalC*, *nalD*, and *mexR* which are all negative regulators of *mexAB*-*oprM* as well as in *parS* (Wardell et al. 2019; Fuhs et al. 2024). Also similar to our results, when exposed to a combination of antibiotics, mutations are commonly found in *mexR*, *nalC*, or *nalD* that all encode negative regulators of *mexAB*-*oprM* and *parS*/*parR* encoding a positive regulator of *mexXY* and *mexEF*-*oprN* (Barbosa et al. 2020).

There are also cases where our results differ from in vitro or in vivo observations. For instance, in vitro evolution in response to TOB is dominated by mutations in *fusA1*, or genes related to lipopolysaccharides synthesis/modification and the electron transport chain (Sanz-García et al. 2018a; Laborda et al. 2022). Since these processes are not included in our regulatory network model, we were unable to identify any associated mutational targets. There are also cases in which genes present in our modeling framework were less often identified as mutational targets than in empirical settings. For example, *algU* appears rarely (<15%) in our simulations in response to CIP. Yet, in empirical settings it is a common mutational target. The reason for the discrepancy may be because *algU* encodes a sigma factor and mutating it can modify alginate production and various stress responses (Hershberger et al. 1995; Folkesson et al. 2012). Our modeling framework also over predicted some mutational targets, eg mutations in *ampR*, *mvaT*, and *rocS2* are not commonly found in in vitro experiments and mutations in *ampR* and *vqsM* are not found in in vivo data. These differences could be due to the role of some of these targets as global regulators, which could cause mutations to have negative pleiotropic effects.

Our modeling framework can also be used to predict interactions between antibiotics, such as cross-resistance. In our simulations, evolution in the presence of one antibiotic frequently resulted in cross-resistance to multiple other antibiotics, similar to in vitro studies (Barbosa et al. 2020; Sanz-García et al. 2018a; Laborda et al. 2022; Barbosa et al. 2017; Sanz-García et al. 2018b). In some sense, this could be predicted in the absence of any simulations, simply based on the fact that efflux pumps can often act on multiple antibiotics. Thus, increasing the expression of a pump will likely lead to an increased ability to remove several antibiotics. However, modeling and simulations can reveal the role of expression levels in shaping cross-resistance patterns. For example, we found that cross-resistance can differ depending on the relative expression level of efflux pumps. When all pumps were equally expressed (Case A) cross-resistance after TOB exposure was much more frequent than when *mexAB*-*oprM* had higher relative expression (Case B). Thus, even with the same efflux pumps and the same regulatory network architecture, cross-resistance may depend on the actual expression levels of pumps. These results indicate a benefit in explicit forecasting models that can be tuned to incorporate gene expression information.

Another type of interaction between antibiotics is collateral sensitivity. We found very few instances of collateral sensitivity in our model. It mainly occurred after evolution in response to MER, causing increased susceptibility to TOB. Similar instances of collateral sensitivity have also been reported in in vitro studies where mutants with increased resistance to beta-lactams (including carbapenems like MER) are more susceptible to aminoglycosides like TOB (Hernando-Amado et al. 2020; Barbosa et al. 2017). In our model, collateral sensitivity occurs due to downregulation of *mexXY*, which increases availability of the shared OprM protein for MexAB-OprM—the only pump that removes MER. There are other examples of collateral sensitivity from empirical observations that our modeling framework did not identify. For instance, in vitro studies have found increased susceptibility to aztreonam and colistin after CIP resistance evolution (Hernando-Amado et al. 2022) and increased susceptibility to MER and TOB in *nfxB* mutants (Mulet et al. 2011). This mismatch between our model predictions and empirical data suggests that important regulatory connections may be missing, but once identified they can be included in our model to refine future forecasts.

The fitness of resistant mutants in the absence of antibiotics is a key trait that determines their ability to spread in the environment and between patients in competition with susceptible strains. While fitness costs are common, they can also quickly be reduced by either reverting mutations or by compensatory mutations. Importantly, in cases where deletions have resulted in loss of negative regulators, the mutations cannot be directly reverted and proper regulation is lost resulting in high constitutive production of costly efflux pumps. Compensating for this dysregulation could then result in further degradation of regulatory networks and efflux pumps. This process could potentially be harnessed to guide bacterial populations into evolutionary dead ends, where they have reduced ability to re-evolve resistance or low fitness due to loss of key genes. We used our model to further explore these questions. After in silico evolution in the presence of antibiotics, we find, as expected, that production of efflux pumps is increased, which leads to increased protein production costs. These costs remain when antibiotics is removed, and we explored how resistant mutants can evolve to reduce these costs. When allowing only mutations in regulators we find a pattern in deletion of positive regulators and duplication of negative regulators. However, in many cases negative regulators, like *nalD* for MER and TOB, were already deleted when evolving resistance thereby preventing future mutations and restricting the possible evolutionary trajectories. In the case when we also allow mutation in the genes encoding the efflux pumps, we find that they are commonly deleted, indicating a possibility to guide evolution toward genotypes that do not contain the efflux pump genes, and hence will lack the ability to reacquire resistance by regulatory mutations. Deletion of efflux pumps genes has been observed in in vitro experiments both in the absence of antibiotics (Hernando-Amado et al. 2025) or with antibiotics (Fuhs et al. 2024; Sanz-García et al. 2018b; Wardell et al. 2021) where collateral sensitivity can also select for loss. Interestingly, mutations in efflux pump genes are also among the most commonly mutated genes during in-patient evolution (Marvig et al. 2014) (Table S1) and deletion of efflux pump genes has even been observed during short-term treatment (Wheatley et al. 2021). In cases where these mutations are irreversible, ie large deletions, the reduced potential for the bacteria to re-evolve resistance could be used to inform design of treatment protocols.

In our model, fitness costs arise solely from protein production and from antibiotic stress, with efflux pumps assigned a 3-fold higher biosynthetic cost than regulators to reflect their tripartite structure. This simplifying assumption preserves the expected higher cost of producing efflux pumps compared to regulators. However, efflux pumps also impose an energetic “running cost,” using the proton-motive force (PMF) for active transport, and these costs are not included in the model. This most closely represent conditions in which generation of PMF is not limiting growth, as is common under aerobic respiration. However, the energetic burden of pump activity interacts in complex ways with cellular metabolism, environmental conditions, and physiological state. For example, during slow growth, PMF-dependent running costs may dominate because protein synthesis rates are low, increasing selection for loss of efflux pump genes once they have been upregulated by mutation. Pump-associated costs will vary substantially depending on whether cells rely on aerobic or anaerobic respiration or fermentation, as well as on environmental factors such as extracellular pH. Moreover, mutations can interact epistatically when multiple pumps or processes use a shared, limited PMF or for mutations that reduce PMF, including electron transport chain mutations that lead to reduced aminoglycoside uptake. While being difficult to model, these kinds of interactions might play a major part in determining the evolutionary trajectories of efflux mutants and provide additional ways use resistance-fitness trade-offs to guide evolution.

Our modeling framework demonstrates the extent to which evolutionary adaptations can be predicted even in the absence of complete empirical data. In developing our model, we necessarily adopted a number of simplifying assumptions, eg constant transcription and translation rates across genes as well as equal rates of mutations across genes. Yet, despite these simplifications, the model successfully reproduced evolutionary patterns observed both in vitro and in clinical settings. An important part of our approach was the use of genetic algorithms and stochastic evolutionary simulations to explore an ensemble of parameterized networks. By aggregating results across many versions of a network, it reduced the sensitivity to specific parameter choices and instead revealed the predictive power of regulatory structure. The availability of additional information—eg expression level differences between planktonic and biofilm growth—could also be incorporated to refine the scope of predictions. The flexibility of this modeling framework allows for the exploration of other possibilities, such as the effects of applying different antibiotics in various sequences. Predictions of the model can then be used to focus experiments on gaining information with predictive power. Repeated iterations of modeling predictions and empirical validations/observations could harness the patterns of antibiotic resistance to create effective evolutionarily designed treatments.

## Methods

### Protein concentrations inside cells

Gene expression can be modeled as a two-step dynamical process (Hargrove and Schmidt 1989) with variables describing the concentrations of mRNA (M) and protein (P):


$$\begin{array}{rl} \frac{dM}{dt} & =K_{m}-\Phi_{m}M\\ \frac{dP}{dt} & =K_{p}M-\Phi_{p}P. \end{array}$$

Here, $K_{m}$ and $K_{p}$ are the transcription and translation rates, and $\Phi_{m}$ and $\Phi_{p}$ are the decay rates for mRNA and proteins, respectively. The equations give the following steady values for mRNA and protein:


$$M^{*}=\frac{K_{m}}{\Phi_{m}},P^{*}=\frac{K_{p}M^{*}}{\Phi_{p}}=\frac{K_{m}K_{p}}{\Phi_{m}\Phi_{p}}.$$

For $K_{p}>>K_{m}$, the two-step process can effectively be considered as a one-step gene-to-protein creation process with a rate $K_{tr}=K_{p}K_{m}/\Phi_{m}$ and steady-state protein level $P^{*}=K_{tr}/\Phi_{p}$. Thus, if we consider a set of different proteins $P_{i}$ (where *i* is an index) then their abundance can be represented by the set of dynamical equations:


$$\frac{d[P_{i}]}{dt}=K_{tr}G_{i}-\Phi_{p}[P_{i}],$$

where $G_{i}$ is the copy number of gene *i*.

Applying this model formulation to *P. aeruginosa*, we can estimate the values of the various rates. The average mRNA and protein levels (per gene) in a *P. aeruginosa* cell are approximately $M^{*}=0.01$ and $P^{*}=200$ (Zhang et al. 2023). The decline in protein levels is mainly caused by dilution during cell division (Hausser et al. 2019). Since *P. aeruginosa* has a doubling time of 2.3 h (Gibson et al. 2018), the mean rate of protein decay is approximately $\Phi_{p}=ln(2)/2.3∼0.3\;h^{-1}$, and the rate of gene to protein creation can be estimated as $K_{tr}=P^{*}\Phi_{p}=60\;h^{-1}$. To model the action of regulatory proteins that bind to promoter regions and either block or catalyze transcription, the equation is modified as (Mendes et al. 2003):


(1) $$\begin{array}{rl} \frac{d[P_{i}]}{dt} & =K_{tr}\prod_{j}\left(\frac{1}{1+K_{ij}^{-}[P_{j}]}\right)\\ & \;\times \prod_{j}\left(1+\frac{K_{ij}^{+}[P_{j}]}{1+K_{ij}^{+}[P_{j}]}\right)G_{i}-\Phi_{p}[P_{i}], \end{array}$$

where $K_{ij}^{+}$ and $K_{ij}^{-}$ indicates the strength of regulation of gene *i* by protein *j* (+ sign is for positive regulators and − sign is for negative regulators).

### Fitness costs

Fitness in our model system depends on two costs experienced by cells: the cost of protein production and the cost of antibiotics. To estimate both of these costs, we assume that the dynamics of the system in Equation (1) are sufficiently fast that we can use the steady state concentrations of proteins. For a given antibiotic, we group together all pumps that target it and call the sum of their steady state values $P_{\mathrm{pump}}$. Since, each efflux pump consists of three proteins, and they are transcribed together, the number of efflux pumps ($P_{\mathrm{pump}}$) in a cell can be considered to be equal to the number of either mex ($P_{mex}$) or opr ($P_{opr}$) proteins. The exception is the MexXY pump with two components which needs OprM sharing from the MexAB-OprM pump. So, we modeled the number of MexAB and MexXY pumps as,


$$\begin{array}{rl} P_{\mathrm{pump}}^{mexAB} & =P_{oprM}\times \frac{P_{mexA}}{P_{mexA}+P_{mexX}}\\ P_{\mathrm{pump}}^{mexXY} & =P_{oprM}\times \frac{P_{mexX}}{P_{mexA}+P_{mexX}}. \end{array}$$

We group the remaining proteins together and call the sum of their steady state values $P_{\mathrm{rest}}$. The cost of protein production ($C_{P}$) is then:


(2) $$C_{P}=P_{\mathrm{pump}}+P_{\mathrm{rest}}.$$

The cost of antibiotic ($C_{A}$) may depend on many factors including the duration exposed to antibiotics and the efficacy of the pumps. For simplicity, we assume that the cost of antibiotics is inversely proportional to the specific pump(s) that pump out a particular antibiotic $P_{\mathrm{pump}}^{\mathrm{relevant}}$, ie


(3) $$C_{A}∼\frac{1}{P_{\mathrm{pump}}^{\mathrm{relevant}}}.$$

Thus, if there are more pumps the cost of antibiotics will be less because they are actively being removed from cells.

## Supplementary Material

msag065_Supplementary_Data

## Data Availability

The main codes used to generate the results in the manuscript are available at this Github link.
