# Supplementary material for: Forecasting Multitrait Resistance Evolution under Antibiotic Stress
Source: Mol Biol Evol. 2026 Mar 13;43(4):msag065. doi: 10.1093/molbev/msag065 (PMC13049199; doi:10.1093/molbev/msag065)
Supplement: msag065_Supplementary_Data [file msag065_supplementary_data.pdf]

Supplementary Information

Genetic algorithm to estimate strengths of regulation and the robustness of those values

The strengths of regulation $K^{\pm}_{ij}$ for the gene regulatory network are unknown. Therefore, we developed a genetic algorithm to estimate these parameters by fitting the $K^{\pm}_{ij}$ adjacency matrix in a way that generates some desired steady state values of the efflux pump proteins following Equation-1 of main text. We considered two distinct expression scenarios:

- **A:** $P^*_{mexAB} = P^*_{mexXY} = P^*_{mexCD} = P^*_{mexEF}$
- **B:** $P^*_{mexAB} = 5 \times (P^*_{mexXY} = P^*_{mexCD} = P^*_{mexEF})$

The algorithm operates on an adjacency matrix $K^{\pm} = D$, where $D_{ij} > 0$ denotes positive regulation and $D_{ij} < 0$ denotes repression. The fitting process minimizes the sum of squared errors between the simulated steady-state levels (following Equation-1 of main text) of four pump genes and their target values. For example, the error in Case (B) is given by:

$$E = (200 - P_{mexA})^2 + (40 - P_{mexX})^2 + (40 - P_{mexC})^2 + (40 - P_{mexE})^2$$

The algorithm proceeds as follows:

1. Initialization: Start with an adjacency matrix D with initial regulation strengths of ± 0.005.
2. Iterative perturbation: Traverse all non-zero interactions in random order. For each d_{ij}, add a small random perturbation uniformly drawn from [-0.002,0.002].
3. Constraint enforcement: Reject any perturbation that would change the sign of d_{ij} (i.e., convert an activator to a repressor or vice versa).
4. Accept the perturbation only if it does not increase any of the four individual squared errors. This strict criterion ensures simultaneous convergence toward all target expression levels.
5. Termination: Repeat until the total error $E \leq 4$, equivalent to an average error of ~ 1 protein copy per target.

We repeated the fitting process 25 times for each case, generating an ensemble of parameter sets that all satisfy the target expression levels. When we compared the difference between each pair of sets (Figure-S1), we found that each trial generated a different fit. It indicates that networks with different strengths of regulation can cause similar expression levels for the efflux pump genes. Figure-S2 shows the boxplots of the strengths of regulation for different regulatory connections in the network.

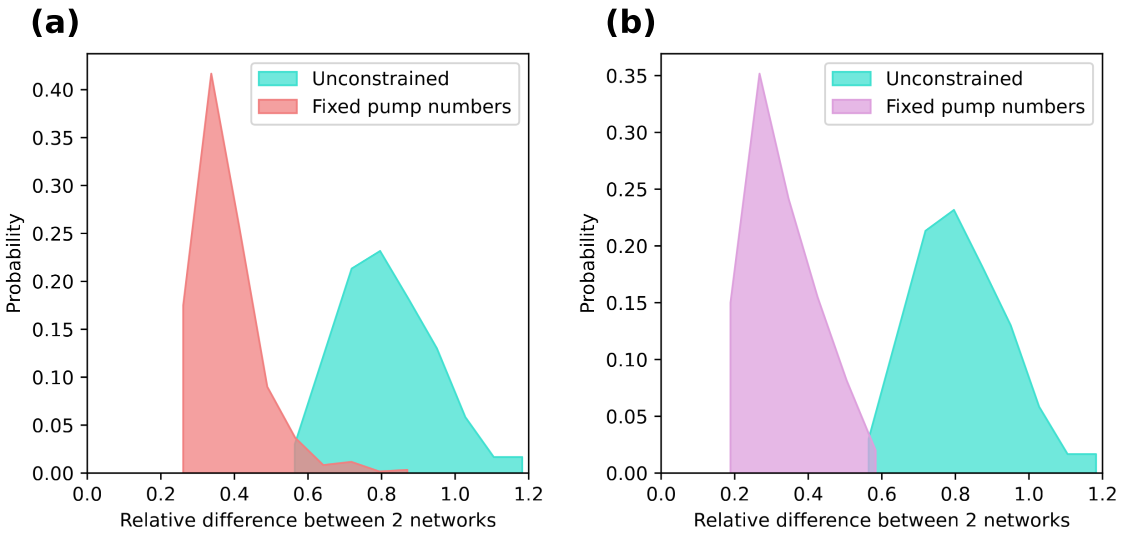

Figure S1: Diversity of the estimated strengths of regulation, generated using the genetic algorithm. **(a):** This figure shows the histogram of the relative difference between 2 different sets of estimates, for Case (A) when all 4 pumps have same expression levels. **(b):** This figure shows the histogram of the relative difference between 2 different sets of estimates, for Case (B) when MexAB pump has higher expression level compared to the other pumps. For comparison, each figure also contains a case when the genetic algorithm is applied without any constraint on the expression level of any gene. The figures show that there can moderate level of diversity for the strengths of regulation, that can generate a similar expression levels for the efflux pumps.

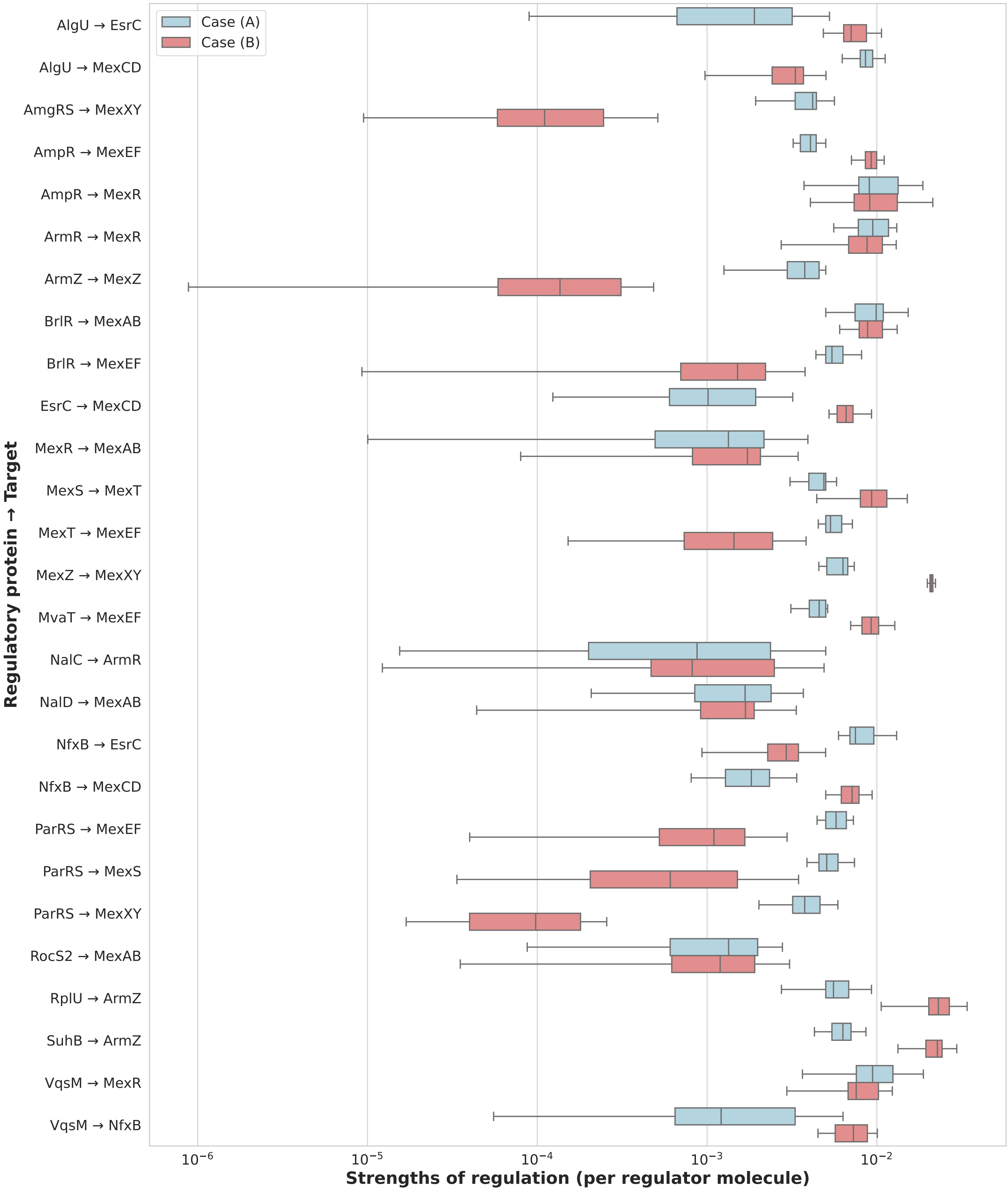

Figure S2: The estimated strengths of regulation, generated using the genetic algorithm. This figure shows the boxplots of the strengths of regulation for each regulatory link. The boxplots are generated using different trial runs of the genetic algorithm. The figure shows the strengths for both Case (A) when all 4 pumps have same expression levels and Case (B) when MexAB pump has higher expression level compared to the other pumps. The x-axis in this figure is in log scale.

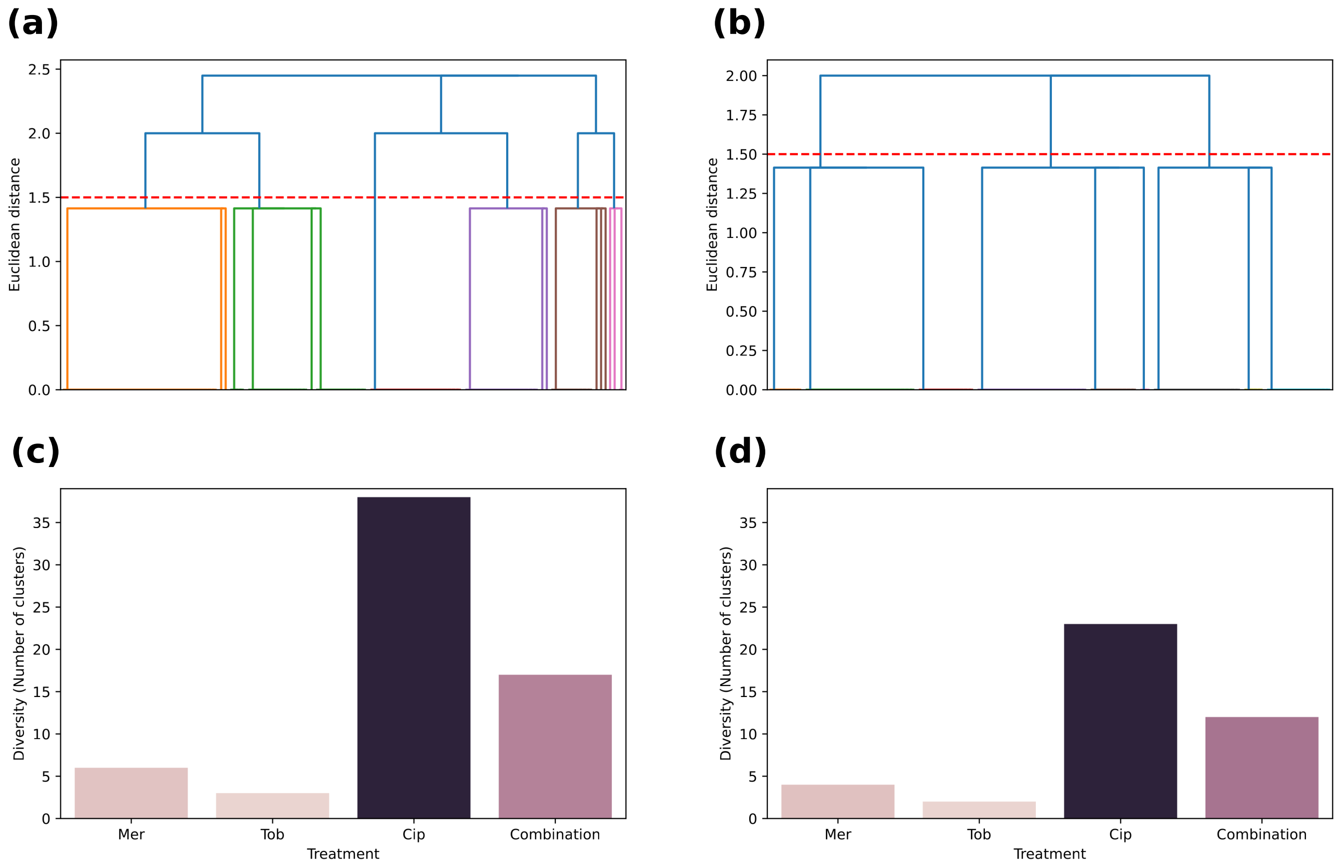

Figure S3: Mutational diversity under different antibiotic treatments. **(a)** and **(b):** These figures show 2 examples of the hierarchical clustering dendrograms, for the mutated genomes following meropenem and tobramycin treatments respectively (in Case A when all pumps have same expression levels). We chose the threshold value to be 1.5 for counting the number of clusters. **(c):** This bar plot shows the diversity of mutated genomes (measured in terms of the number of clusters) for Case A. **(d):** This bar plot shows the diversity of mutated genomes for Case B, when the MexAB pump has higher expression level. The figure shows that evolution under ciprofloxacin treatment can lead to a very high mutational diversity.

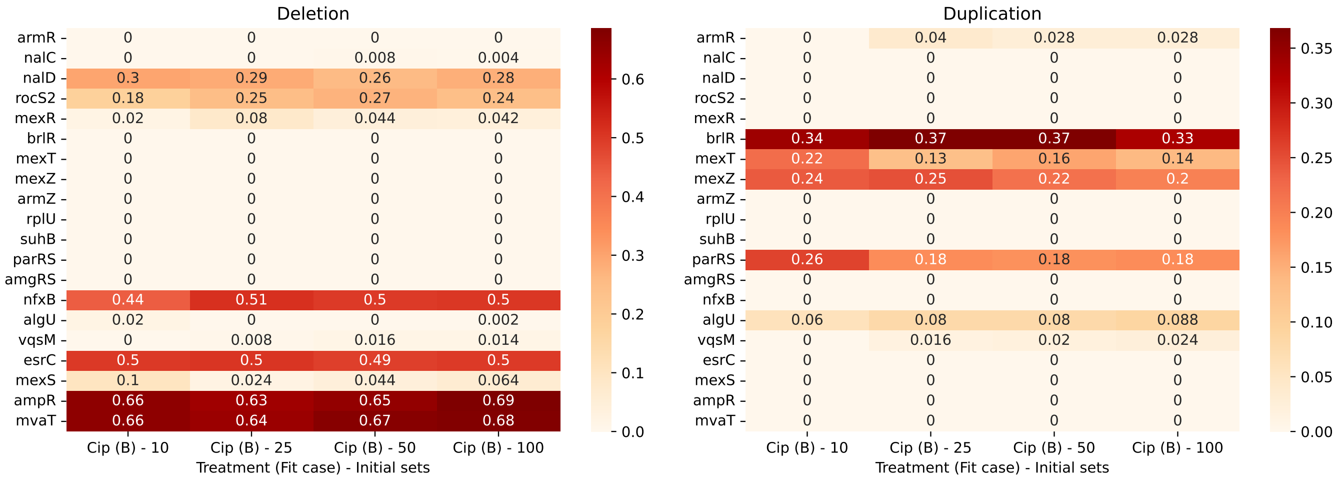

Figure S4: Mutation frequencies of different genes under ciprofloxacin treatment Case (B). The heatmap shows the mutation frequencies when the simulations started from 10, 25, 50 and 100 initial parameter sets. The differences in the frequencies become negligible when the number of initial sets is 25 or higher. Thus 25 initials sets can be considered a reasonable sample size.

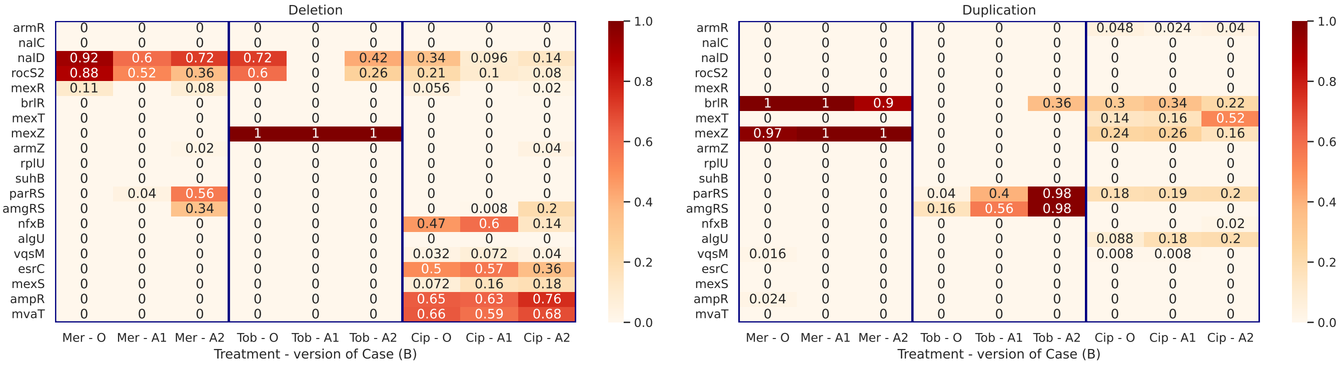

Figure S5: Mutation frequencies of different genes when the Case (B) is constructed in 3 different ways. The heatmap shows deletion and duplication frequencies of the genes under meropenem, tobramycin and ciprofloxacin treatments. The **O** following each treatment indicates the original choice of expression levels for Case (B) i.e. $MexAB = 200, MexXY = MexCD = MexEF = 40$. **A1** denotes first alternate choice: $MexAB = 333, MexXY = MexCD = MexEF = 67$ (symmetrical around average 200, while still maintaining the ratio of the original choice). **A2** denotes second alternate choice: $MexAB = 300, MexXY = MexCD = MexEF = 200$ (MexAB expression is increased instead of reducing the expression of other pumps).

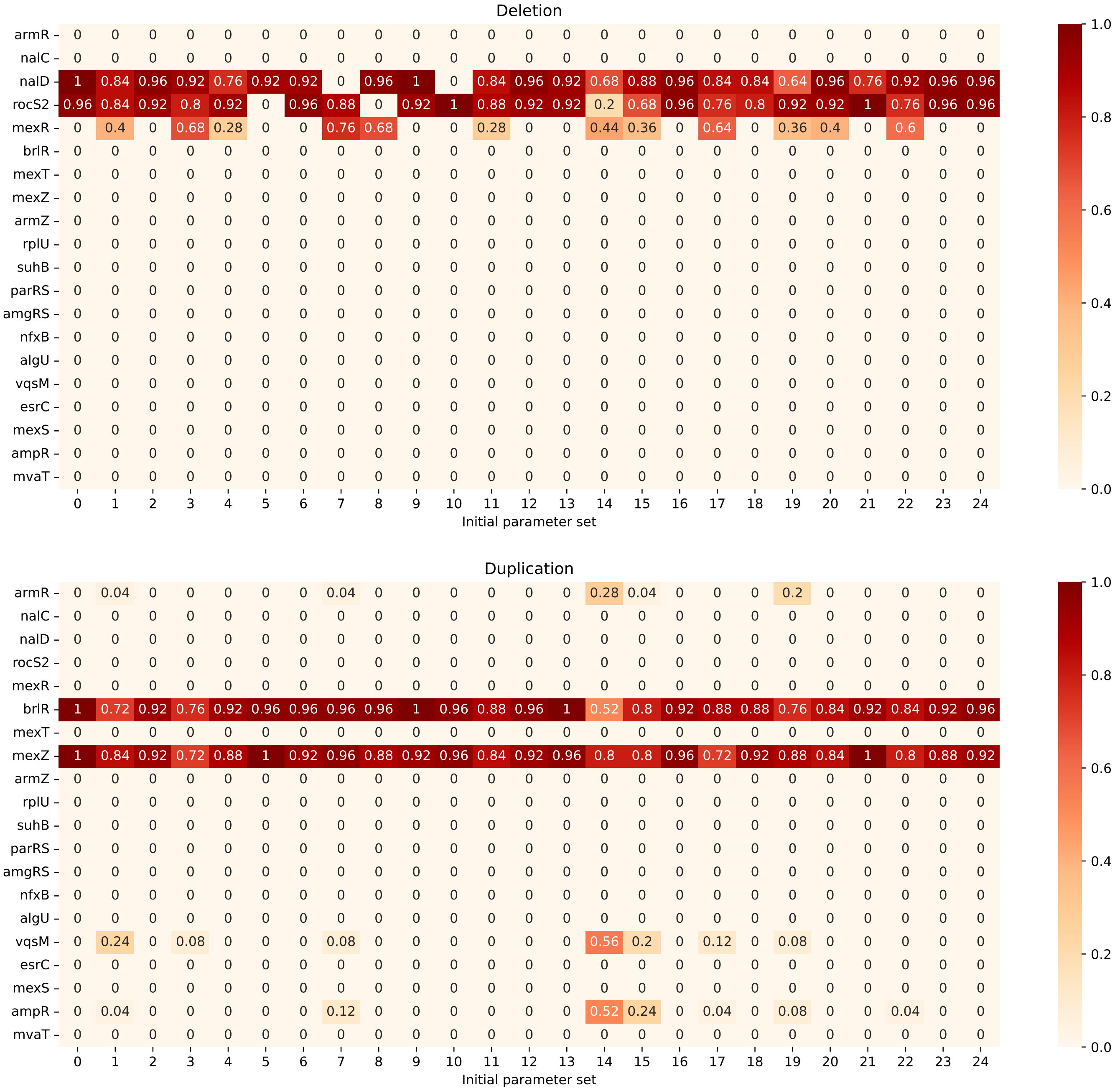

Figure S6: Mutation frequencies of different genes calculated separately for each initial set, by averaging over 25 trials per set. This heatmap shows the mutation frequencies under meropenem treatment for Case (B) (MexAB pump has higher expression than others). The mutation frequencies generally remain consistent between different sets. However some deviations are observed, for example in the case of mexR gene.

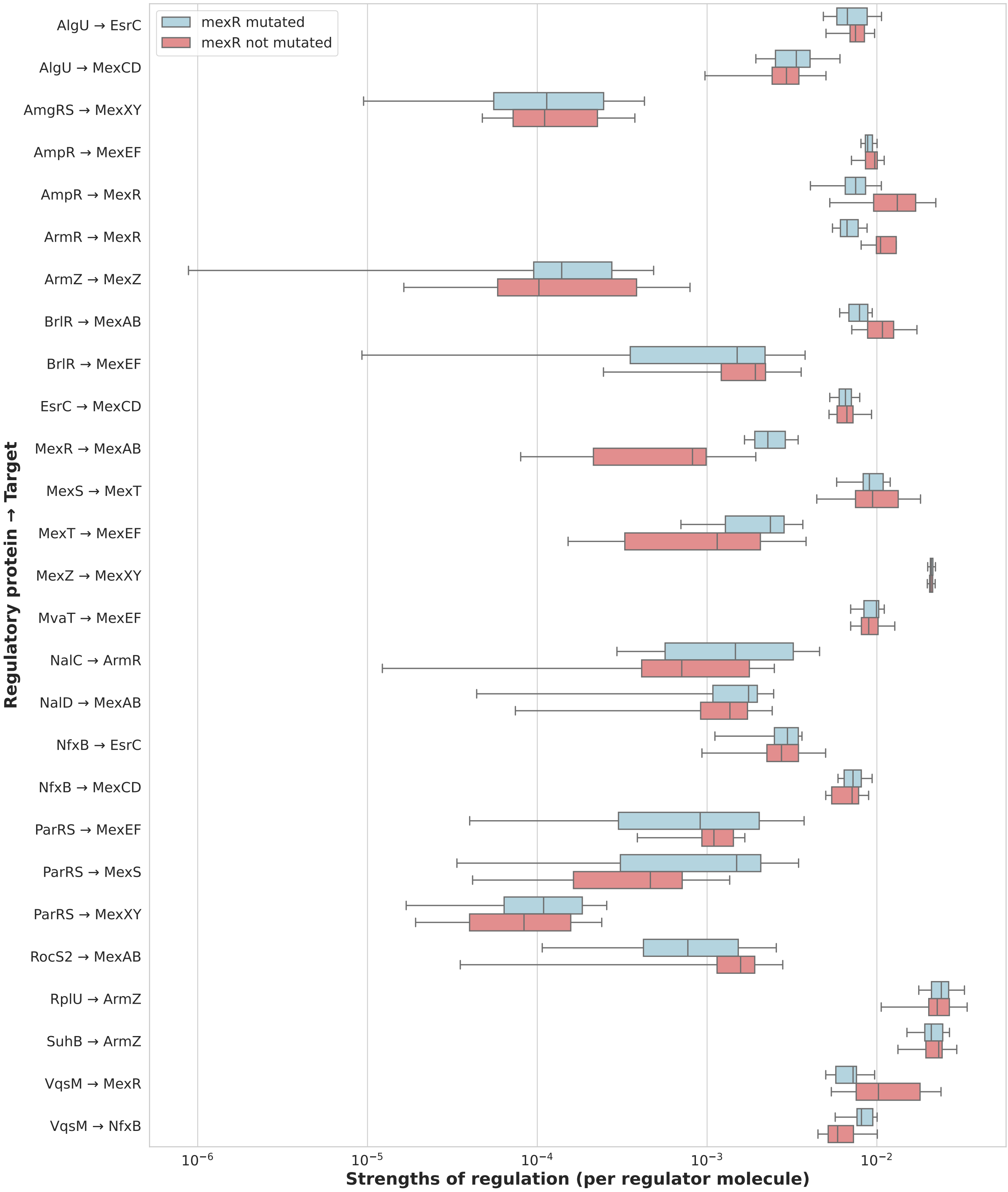

Figure S7: The strengths of regulation for initial sets where mexR is mutated (blue) vs sets where it is not (red). This figure shows the boxplots of the strengths of regulation for each regulatory link. The x-axis in this figure is in log scale. Between these two collections of sets (red and blue), differences in the strength of regulation are observed mainly for the links involving the mexR gene. This indicates the existence of at least two local minima in the parameter space that lead to mexR being mutated or not in response to meropenem.

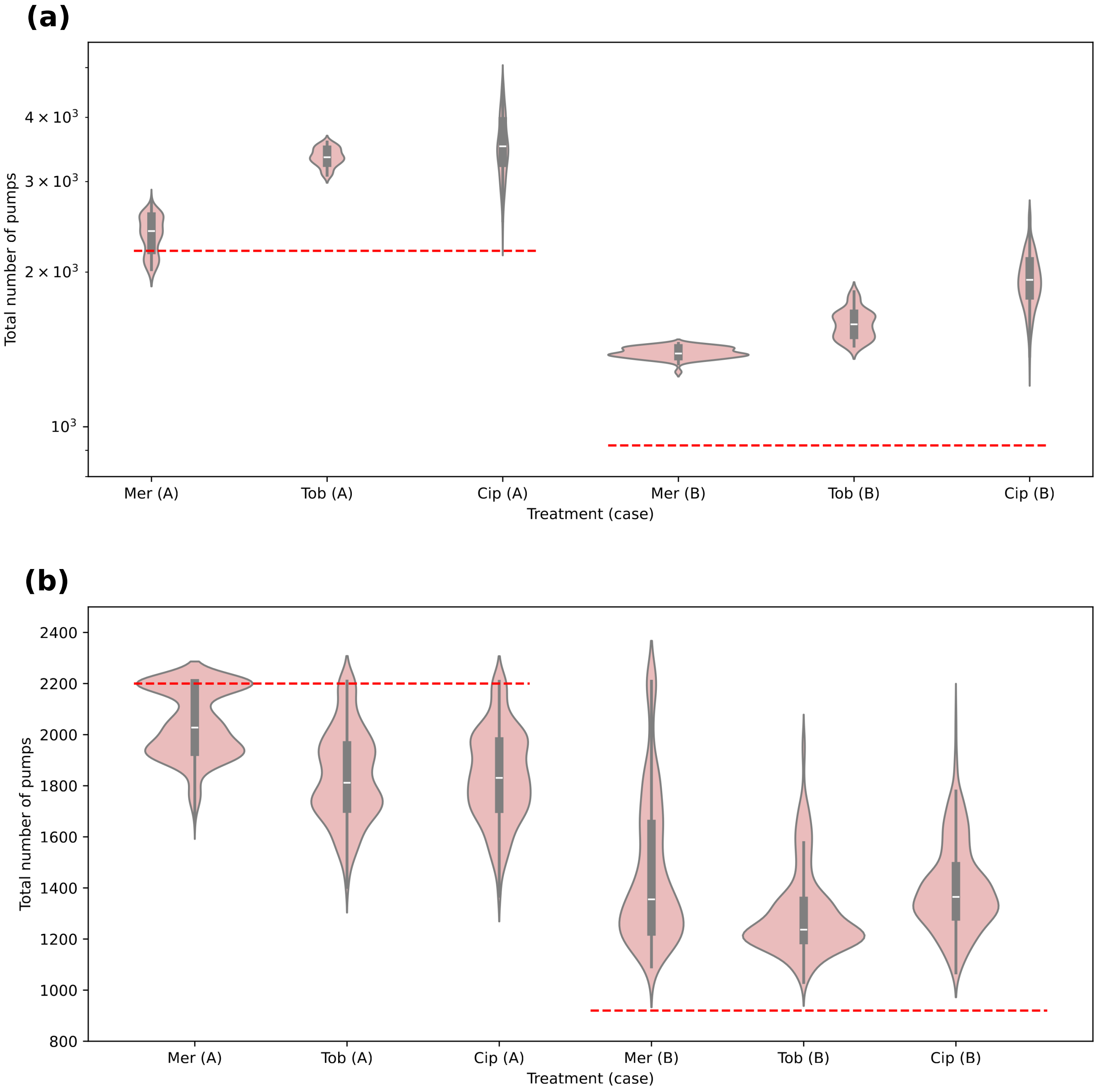

Figure S8: The total number of efflux pumps following different treatments. **(a):** This violin plot shows the number of pumps in the resistant mutants (following treatments) compared to their initial values. Left panel and right panels represent Case A and Case B of initial pump expressions respectively. **(b):** This violin plot shows the number of pumps after the antibiotics are removed and the cells are allow to acquire more mutations in their attempt to go back to the original pump levels. This figure shows that it is not possible to return to the original pump levels in Case B, with mutations in the regulatory genes only.

Table S1: Number of unique mutations during within-host evolution in longitudinally collected clinical isolates of *Pseudomonas aeruginosa* sampled from individuals with cystic fibrosis (from (Marvig et al. 2014)).

Gene	Locus Tag PAO1	Unique SNPs	Unique Indels	Upstream intergenic	
ampR	PA4109	0	0	0	0
armR	PA3719	0	0	0	0
vqsM	PA2227	0	0	0	0
mexX	PA2019	1	0	0	1
rocA2	PA3045	1	0	0	1
brlR	PA4878	1	0	0	1
suhB	PA3818	0	1	0	1
mvaT	PA4315	1	0	0	1
oprM	PA0427	1	1	0	2
mexY	PA2018	2	0	0	2
mexE	PA2493	1	1	0	2
armZ	PA5471	1	1	0	2
parR	PA1799	0	0	2	2
oprN	PA2495	3	0	0	3
rocS1	PA3946	3	0	0	3
amgR	PA5200	1	1	1	3
oprJ	PA4597	4	0	0	4
esrC	PA4596	0	0	4	4
rplU	PA4568	0	0	4	4
nalD	PA3574	0	6	0	6
rocS2	PA3044	3	3	0	6
mexC	PA4599	4	0	3	7
mexF	PA2494	5	2	0	7
amgS	PA5199	6	1	0	7
mexR	PA0424	1	7	0	8
nalC	PA3721	3	4	1	8
parS	PA1798	10	0	0	10
mexS	PA2491	9	1	0	10
mexA	PA0425	3	9	0	12
mexT	PA2492	9	5	1	15
mexD	PA4598	17	0	0	17
nfxB	PA4600	10	8	3	21
mexB	PA0426	6	16	0	22
algU	PA0762	22	6	2	30
mexZ	PA2020	10	30	0	40

References

Marvig, Rasmus Lykke et al. (Nov. 2014). "Convergent evolution and adaptation of Pseudomonas aeruginosa within patients with cystic fibrosis". In: *Nature Genetics* 47.1, pp. 57–64. ISSN: 1546-1718. DOI: 10.1038/ng.3148. URL: http://dx.doi.org/10.1038/ng.3148.
